# Diseases of the Eye and Ear

**Published:** 1890-09

**Authors:** 


Diseases of the Eye and Ear. By C. H. Vilas, A. M., M. D., Professor of Diseases of the Eye and Ear in the Hahnemann Medical College, Chicago. For sale by Bœricke & Tafel.

This work, containing much that is of practical value, like many others coming from hands which claim to be homœopathic, is marred by antipathic recommendations in the treatment of various affections of the eyes and ears. Thus, at p. 8, we find: "The treatment of eye diseases demands the use of solutions prepared from various alkaloids. First among such, and without which the treatment of eye diseases would be extremely hazardous, is atropia sulphate, commonly called atropine." We say, unqualifiedly, this is incorrect. The simillimum is all that is necessary in any disease of the eye—or in disease in any other organ. Disease is not in the eye; it is in the system, and can only be overcome by proper systematic treatment. This is mere platitude, or should be, for it has sufficient age to make it common. The idea of a teacher in a college bearing the name of Hahnemann giving expression to such teachings—ideas which one hundred years ago might have gone unquestioned. But Hahnemann has lived since then! and he has followers who are ever ready to deny such teachings. One can get some valuable hints from Dr. Vilas's book; but he had better avoid the allopathic treatment advised. G. H. C.

